# Dermatology

**Published:** 1909-06

**Authors:** J. A. Nixon


### DERMATOLOGY.

**Sporotrichosis** as a clinical entity has only lately come to be recognised as comprising a large group of lesions dependent upon a definite infection comparable to granulomata, such as syphilis, tuberculosis, and actinomycosis.

In England little has been heard of the disease, perhaps owing to its extreme rarity ; but in France de Beurmann and others have devoted much time and pains to the separation of this condition from those with which until 1902 it had always been confused and grouped.

The polymorphism of sporotrichosis is equal to that of either syphilis or tuberculosis. As de Beurmann writes : "Sporotrichosis starts in the great majority of cases without affecting the general health, without fever, gastro-intestinal troubles, or wasting ; the disease confines itself to cutaneous or subcutaneous localisations. . . . The lesions in or beneath the skin evolve slowly and coldly. . . . Still, all the observations do not agree with this usual type of a chronic and torpid affection. There are sporotrichoses with a sudden onset marked by general disturbance and fever ; as in acute infections the appearance of the lesions is rapid and simultaneous, constituting a true eruption. Others, after an onset more or less sudden, continue under the form of febrile attacks, with profound interference with the general health, gastro-intestinal troubles, wasting, etc., each recrudescence of the disease being accompanied by a fresh gummatous eruption. Others run a subacute course, with wasting and severe anæmia. In all these cases the mycosis behaves like an acute or subacute general infection ; it resembles the coccal septicæmias with cutaneous metastases." Such, briefly, is the epitome of de Beurmann.[1]

The first cases recognised were published in America by Schenck,[2] and by Hektoen and Perkins,[3] under the title of

[1] De Beurmann and Gougerot, *Ann. de Dermat. et Syph.*, 1909, x, 83.
[2] Schenck, *Bull. Johns Hopkins Hosp.*, 1898, ix, 286 (with plates).
[3] Hektoen and Perkins, *J. Exper. M.*, 1900, v. 77 (with plates).

"Refractory subcutaneous abscesses caused by sporothrix Schenckii. A new pathogenic fungus," a description given by Schenck which has been wholly confirmed by subsequent observations. This previously unknown disease, which had long been confounded with tuberculous lesions and with those of heredo-syphilis, aroused great interest.

The first three cases described were due to obvious local inoculation: a scratch of the skin of the finger by a nail (Schenck), a blow upon the finger by a hammer (Perkins), and puncture of the finger by a piece of wire (Brayton). From this inoculation indolent abscesses resulted, both locally and in the course of the lymphatics, which proved characteristically refractory to surgical treatment, and were ultimately shown to contain a fungus now identified by the name of *sporotrichum Beurmanni.*

Adamson[1] has recently summed up our knowledge of this specific infection, and from his paper the following description is worth quoting: "The cases fall into two main clinical types—the American cases with a primary local infection and a line of chronic abscesses in the arm, connected by a train of chronic lymphangitis. In the second group, the French cases, there were multiple and widely distributed abscesses generally hypodermic, but sometimes associated with these dermic and epidermic lesions."

De Beurmann describes a syphiloid type and a tuberculoid type according as the lesions simulate syphilitic gummata or tuberculosis verrucosa. The lymphatic glands are generally uninvolved.

It has not been clearly demonstrated that the viscera are ever infected, though this seemed probable in one case.

In one case which showed *sporotrichum* in the sputum the lungs were sound. In other cases cutaneous sporotrichosis has occurred in association with pulmonary tuberculosis.

Recently cases have been recorded in which gummata were present, acute or chronic osteitis, periostitis osteomyelitis arthritis and synovitis.[2] Lesions of the mucous membrane have been reported, and also the presence of *sporotrichum* in the bucco-pharynx and larynx without causing lesions.

In the diagnosis the main points to observe are multiplicity of lesions, indolence, slow involution, firm consistence, viscid grey yellow homogeneous pus, swollen margins with central fistulous opening into the cavity, generally absence of enlarged glands, good general health. The crateriform character of the lesions is especially characteristic. In those cases where the base of the exposed cavity is crusted or vegetating, simulating cutaneous tuberculosis, sporotrichosis is suspected from the

1 Adamson, *Brit. J. Dermat.*, 1908, xx, 296 (with Bibliography).

2 De Beurmann, Gougerot and Vaucher, *Rev. de Chirurg.*, 1909, xxix, 4, p. 661 (with plates).

central softening of the lesions, and from the occurrence of more typical gummata elsewhere. Sporotrichosis must be considered, too, in the presence of gummatous periosteal swelling and chronic intra-muscular abscesses.

The diagnosis is readily made by cultural methods, remembering the importance of using suitable media (Sabouraud's peptone-glucose-agar), of not capping the tubes, and of incubating at ordinary room temperature.

In culture, colonies appear on the fourth to sixth day as small white acuminate points 1 mm. in diameter, surrounded by a white areola, finely rayed. They slowly increase in size, and become convoluted and brown in colour. Films from cultures show long filaments 2 $\mu$ broad, together with numerous ovoid spores 5 to 6 $\mu$ in length by 3 to 4 $\mu$ broad. Here and there single spores or bunches of three to thirty are seen attached to the mycelial filament by a short pedicle.[1] De Beurmann has shown that an early orchitis in the rat, after inoculation with suspected products, is diagnostic of sporotrichial infection.

In addition to this description of Adamson, other means of diagnosis may be mentioned. (1) Sporo-agglutination by the serum of patients affected with sporotrichosis,[2] (2) presence of the parasites in the blood,[3] (3) fixation of complement,[4] (4) determination of the opsonic index to the sporotrichum.[5]

As to the habitat of the parasite, de Beurmann and Gougerot have found in two different parts of the French Alps three specimens of the *sporotrichum Beurmanni* as a vegetable parasite.

The histology of the lesions is that of tubercle or syphilis. The lesions present three types of reaction in combination :—

(1) Lympho-connective tissue or syphiloid reaction.
(2) An epithelioid (with giant cells) on tuberculoid reaction.
(3) A polynuclear or ecthymatiform reaction.

The histology of this disease tends to destroy the contention that there is any specific histology of either syphilis or tubercle, for all the changes seen in these diseases are seen here. As de Beurmann and Gougerot say, "There is no such thing as anatomical specificity ; there is only specificity of the causal parasite. The *sporotrichum Beurmanni* and the bacillus of Koch produce an identical nodule."[6]

Of treatment Adamson says : "It is characteristic of the lesions that they are remarkably obstinate to the ordinary surgical methods of treatment. Under the administration of iodides they rapidly disappear."

1 See also *Brit. M. J.*, 1908, ii. Epit., p. 79.
2 Widal and Abrami, *Bull. de la Soc. Med. des Hôp. de Paris*, 1908, 947.
3 Widal and Weill, *Gaz. d. Hôp.*, 1908, lxxxi, 847.
4 Widal and Joltrain, *Ibid.*, 1649. 5 Caussade, *Ibid.*, 920.
6 De Beurmann and Gougerot, abstract in *Brit. J. Dermat.*, 1908, xx, 202.

**The cerebro-spinal fluid in some dermatoses of infancy.**—In a recent article Ravaut called attention to the frequent occurrence of a marked lymphocytosis in the cerebro-spinal fluid of children suffering from heredo-syphilis, who appear to present no nervous symptoms, but show extensive cutaneous lesions. When the mucous and cutaneous lesions are unimportant, the reaction is as a rule slight ; and the reaction is constantly absent among the offspring of syphilitic parents, where there are no cutaneous manifestations. Tobler[1] regarded this lymphocytosis in the cerebro-spinal fluid as being of great value in establishing the fact of heredo-syphilis in apparently healthy subjects.

Ferraud[2] has done valuable work in submitting these views to a critical examination. His researches deal with the cytology of the cerebro-spinal fluid of infants apparently healthy, who showed none of the stigmata or antecedents of heredo-syphilis. He carefully excluded all subjects who presented signs of fever, or symptoms of pulmonary, aural or meningeal disorders capable of setting up any reaction in the spinal fluid.

At the outset Ferraud anticipated that meningeal lymphocytosis would prove to be absent in non-syphilitic dermatoses, and he hoped thus to establish a means of differentiating simple eruptions from those of specific origin when all other signs of syphilis were absent. This expectation failed to be fulfilled, for it was discovered that lymphocytosis was met with far too frequently in certain infantile eruptions to possess any absolute diagnostic importance.

The conclusions reached in the course of 120 observations were as follows :—

The appearance of a cellular reaction, even to a marked degree, in the cerebro-spinal fluid may be a common occurrence.

During the course of certain skin affections in children, especially in the infantile eruptions in prurigo or in scabies, a lymphocytosis of the cerebro-spinal fluid is frequently found. This reaction is inconstant, and only attains to any importance, as a rule, in extensive erythemas (erythema lenticulare) and prurigos. It is quite comparable to the reaction met with in the cutaneous manifestations of syphilis, acquired or hereditary.

In the absence of a previous history or obvious stigmata of syphilis, the existence of meningeal lymphocytosis appears to have only a relative value in the diagnosis of heredo-syphilis. Even in cases where syphilitic antecedents have been observed it is very necessary, if this reaction is to maintain a serious value, that it should be *interpreted*.

Ferraud gives certain hints which may assist in the correct interpretation of cyto-diagnosis of the cerebro-spinal fluid :—

[1] *München. med. Wchnschr.*, 1906, liii, 1890 : abstract in *Bristol M.-Chir. J.*, 1906, xxiv, 348.

[2] Ferraud, *Gaz. d. Hôp.*, 1908, lxxxi, 1539.

1. The lymphocytoses of non-syphilitic eruptions are often very slight, varying from time to time, and are probably less persistent.

2. The cyto-diagnosis not infrequently reveals, in addition to the predominating lymphocytosis, the presence of large mononuclear cells and of endothelial cells in greater abundance than occurs in heredo-syphilis. It is important, moreover, to bear in mind that just as certain previous or co-existent diseases may affect the number of leucocytes in the cerebro-spinal fluid—especially intestinal or pulmonary toxæmias, infections of the middle ear, herpetic eruptions, etc.—so too in young children, particularly nurslings, one must take into account the state of the skin. Once more it is demonstrated that a cellular reaction even of considerable degree may occur quite commonly in the cerebro-spinal fluid; further study may perhaps show certain distinguishing features between these various reactions. But at present we lack sufficient information to entitle us to give to this lymphocytosis a specific value, or to make it a symptom of either tuberculosis or syphilis.

J. A. Nixon.

---

